# Draft Genome Sequence of *Euryhalocaulis caribicus* Strain JL2009^T^, a New Member of the Family *Hyphomonadaceae* Isolated from the Caribbean Sea

**DOI:** 10.1128/genomeA.00407-13

**Published:** 2013-08-08

**Authors:** Yao Zhang, Zihao Zhao, Wenchao Deng, Xiabing Xie, Nianzhi Jiao

**Affiliations:** State Key Laboratory of Marine Environmental Sciences, Xiamen University, Xiamen, China

## Abstract

*Euryhalocaulis caribicus* strain JL2009^T^ is a novel genus and species of the family *Hyphomonadaceae* and was first isolated from surface water in the Caribbean Sea. Here, we report the first draft genome from this genus. Its genome contains genes encoding proteins that are involved in organic acid metabolism and probable low-affinity inorganic phosphate transporters, which suggests its competence in oligotrophic oceans.

## GENOME ANNOUNCEMENT

*Euryhalocaulis caribicus* strain JL2009^T^ (=CGMCC 1.12036 T = JCM 18163^T^), the type strain of *E. caribicus* gen. nov., sp. nov., was originally isolated from surface water in the Caribbean Sea (1). This strain is aerobic, Gram-negative, chemo-organotrophic, and euryhaline and forms a distinct phylogenetic lineage within the family *Hyphomonadaceae* (2). The members of this family have been isolated from a wide range of aquatic environments, involving freshwater and seawater, but most of them were obtained from the oceanic environments. It is believed that *E. caribicus* plays an important role in carbon cycling in its habitats due to the property of dimorphism, which is helpful in competing for resources and resisting an insufficient supply of nutrients for a long time when cells form stalks (3, 4).

To assess the metabolic potentials of the new genus *Euryhalocaulis*, we sequenced the whole genome of *E. caribicus* strain JL2009^T^ using Roche GS FLX+ pyrosequencing technology. A total of 209,097 reads (mean, 393 bases) were assembled using Newbler 2.6 software and produced 39 contigs covering 3,323,095 bp. Contigs of <500 bp long were removed from the final assembly. The average contig length is 155,932 bp and the maximum contig length is 370,916 bp. Genome coverage was ca. 24.7×. The G+C content is 63.94%.

Gene prediction was prepared using Glimmer 3.0 software (5). Annotation of the coding sequences (CDSs) was performed by searching against the KEGG (6), COG, Swiss-Prot, TrEMBL, NR, and GO protein databases. rRNA and tRNA genes were found using rRNAmmer (7) and tRNAscan (8), respectively. A total of 3,232 genes were predicted, with an average length of 931 bp, accounting for 64.57% of the genome. Among all the predicted genes, 3,186 genes are protein-coding sequences and 46 are rRNA sequences. Functional COG categories were assigned for 1,869 (58.66%) proteins. In addition, 1,605 genes (50.37%) were involved in 164 different metabolic pathways.

A comparison with the genome sequences available at RAST (9) showed that *Maricaulis maris* MCS10 (score, 518) is the closest neighbor of *Euryhalocaulis caribicus* strain JL2009^T^. Genes encoding proteins that are involved in organic acid metabolism and probable low-affinity inorganic phosphate transporters were uniquely identified in the genome sequences of the strain JL2009^T^, suggesting that this novel isolate is capable of utilizing a wide range of carbohydrate sources and is better suited for nutrient-limiting environments. Genes encoding ammonium transporters and complexes for inorganic sulfur assimilation and nitrate reductase were also present in the JL2009^T^ genome.

### Nucleotide sequence accession number.

The *E. caribicus* strain JL2009^T^ genome sequence has been deposited at DDBJ/EMBL/GenBank under the accession no. ASJA00000000. The version described in this paper is the first version.
